# Mindfulness and compassion-oriented practices at work reduce distress and enhance self-care of palliative care teams: a mixed-method evaluation of an “on the job“ program

**DOI:** 10.1186/s12904-017-0219-7

**Published:** 2017-07-06

**Authors:** Claudia L. Orellana-Rios, Lukas Radbruch, Martina Kern, Yesche U. Regel, Andreas Anton, Shane Sinclair, Stefan Schmidt

**Affiliations:** 1 Clinic for Psychosomatic Medicine and Psychotherapy, Medical Faculty, Medical Center, University of Freiburg, Freiburg, Germany; 2 Institute for Transcultural Health Studies, European University Viadrina, Frankfurt (Oder), Germany; 3 Palliative Care Centre, Malteser Hospital Bonn/Rhein-Sieg, Bonn, Germany; 4 Department of Palliative Medicine, University Hospital Bonn, Bonn, Germany; 5 Institute for the Investigation of Border Areas of Psychology and Mental Health, Freiburg in Breisgau, Germany; 6 Faculty of Nursing, University of Calgary, Calgary, AB Canada

**Keywords:** Self care, Burnout, Compassion, Mindfulness, Palliative care, Psychological distress, Qualitative methods

## Abstract

**Background:**

Maintaining a sense of self-care while providing patient centered care, can be difficult for practitioners in palliative medicine. We aimed to pilot an “on the job” mindfulness and compassion-oriented meditation training for interdisciplinary teams designed to reduce distress, foster resilience and strengthen a prosocial motivation in the clinical encounter.

**Methods:**

Our objective was to explore the feasibility and effectiveness of this newly developed training. The study design was an observational, mixed-method pilot evaluation, with qualitative data, self-report data, as well as objective data (cortisol) measured before and after the program.

Twenty-eight staff members of an interdisciplinary palliative care team participated in the 10-week training conducted at their workplace.

Measures were the Perceived Stress Questionnaire, the Maslach Burnout Inventory, the somatic complaints subscale of the SCL-90-R, the Emotion Regulation Skills Questionnaire, the Hospital Anxiety and Depression Scale, and a Goal Attainment Scale that assessed two individual goals. Semi-structured interviews were employed to gain insight into the perceived outcomes and potential mechanisms of action of the training. T-tests for dependent samples were employed to test for differences between baseline and post-intervention.

**Results:**

Significant improvements were found in two of three burnout components (emotional exhaustion and personal accomplishment), anxiety, stress, two emotional regulation competences and joy at work. Furthermore, 85% of the individual goals were attained. Compliance and acceptance rates were high and qualitative data revealed a perceived enhancement of self-care, the integration of mindful pauses in work routines, a reduction in rumination and distress generated in the patient contact as well as an enhancement of interpersonal connection skills. An improvement of team communication could also be identified.

**Conclusions:**

Our findings suggest that the training may be a feasible, effective and practical way of reducing caregiver-distress and enhancing the resources of palliative care teams.

## Background

The challenging demands experienced by palliative care practitioners go hand in hand with experiences of personal fulfillment and high job satisfaction [1]. Contrary to what one would intuitively think about the work with the dying, palliative care practitioners have reported that frequent exposure to death can help them to live in the present, enhance meaning, cultivate a spiritual life and develop curiosity about the continuity of life [2].

Yet, inherent stressors pertaining to the work in this field, can impact the health and well-being of practitioners leading to stress, burnout, psychological morbidity and compassion fatigue [3–5]. Moreover, external factors including the limited healthcare resources, increased clinical demands and negative workplace cultures, can hinder the delivery of compassionate medicine [6].

A national survey conducted in Germany [5] assessing burdening factors experienced by palliative care practitioners (*n* = 873) revealed that 51% of the surveyed practitioners feel strongly or very strongly burdened when they are unable to achieve the objectives of palliative care (e.g. meeting psychosocial needs), 47,2% identified burden related issues regarding relationship building with patients and their families (e.g. balancing closeness and distance) and 42,6% identified frequent patients deaths as a burdening factor. Similarly, a Canadian study with 42 nurses providing end-of-life care in intensive care units, found that in addition to organizational and professional factors, emotional factors represented a significant source of distress in palliative care. Difficulties in managing emotions arise for example through moral distress and frequent exposure to suffering [7]. These results emphasize the specific need for self-care interventions, while also highlighting the importance of addressing relational skills since this seems to remain a significant issue in palliative care [8].

In general, establishing professional boundaries has been recommended to healthcare providers as a mitigator of work-related emotional distress [9, 10]. However, relying solely on self-protection strategies can also strain well-being over the long-term [11, 12]. In fact, a recent study investigating the prevalence of posttraumatic stress disorder symptoms, depression and coping revealed that practitioners who are prone to avoidant coping strategies may have a higher risk for developing posttraumatic stress disorder symptoms [13].

Likewise, a separate study suggests that the quality of care may be compromised through detachment and lack of support [14]. In this sense, an intervention which is beneficial to caregivers and patients must accomplish the seeming paradox: Protect caregivers from being overwhelmed when confronted with suffering while at the same time enhancing their ability to be vulnerable and remain present to suffering.

According to Singer and Klimecki an inadequate empathic sharing of the suffering of others can be the cause of *empathic distress.* [15] By this, they mean, an isomorphic state, a self-oriented emotion dominated by efforts to protect oneself from the negative feelings and stress that are vicariously elicited through the empathic resonance with others. By contrast, they define *compassion* as an other-oriented quality, characterized by positive and warm feelings of concern for the suffering of others and a wish to relieve it. In one recent functional plasticity experiment, participants first engaged in a empathic resonance training before receiving a *Loving-kindness* meditation training [16, 17]. The compassion-oriented practice of *Loving-kindness* is a contemplative practice intended to foster feelings of impartial kindness, warmth and benevolence towards the self and others [18]. After the empathy training, participants reported an increase in negative affect as well as increased activity of brain networks associated with negative affect and empathy for pain, while being exposed to the suffering of others via a video compared to a control group. Conversely, the *Loving-kindness* meditation training could reverse these effects by activating neural networks related to positive emotions, affiliation and love, together with an augmentation of positive feelings. The authors concluded that practicing *Loving-kindness* meditation can protect against burnout, while foster feelings of connectedness, altruism and love when confronted with the distress of others [19].

### Mindfulness meditation

The concept of mindfulness, as it is defined in Buddhism, can be described as awareness of the present moment with a certain width of the mind in which one attempts to observe without interfering [20]. In addition to its focus on attention regulation, mindfulness encompasses an orientation towards experience characterized by an attitude of curiosity and acceptance [21]. Prior studies have supported the effectiveness of mindfulness-based interventions in reducing occupational stress, burnout, anxiety and depression, as well as in improving self-compassion and the spiritual well-being of caregivers [22–26]. According to a qualitative study conducted recently, palliative care practitioners recognize mindfulness as an important tool to enhance work sustainability [27]. Explicitly, they stated a need for mindfulness techniques that can be readily implemented during work hours.

### Metta and Tong-len meditation

Two contemplative practices, have been said to be powerful tools for the fostering of a prosocial motivation: *Metta* meditation and *Tong-len* meditation. In both practices, perception of the body and breathing, visualizations and the so called “*Metta-*sentences” are employed to strengthen feelings of connectedness and love, with the broader goal of cultivating a mental attitude and motivation to be of help to others*.*

*Metta* is a Pali word often translated as *Loving-Kindness* into English. In this meditation, the so called *Metta*-sentences (e.g. ‘may person x be free from suffering’), are internally repeated while visualizing light or warmth in the heart area, as well as the person to whom this feelings are directed to. The core of this practice is not the recitation of the phrases but the mindful awareness of the feelings connected to them [28]. Ultimately, the goal behind *Metta* is to expand the attitude of impartial loving-kindness that is typically experienced for close ones to all persons. Therefore, one usually begins practicing *Metta* for oneself, followed by close, neutral and difficult persons, and finally for all human beings.

The word *Tong-len* is a Tibetan word that can be translated as „sending and taking“. *Tong-len* meditation has been suggested to be effective in overcoming natural barriers of a compassionate attitude, even in extreme and difficult situations. For example fear of one’s own suffering or the suffering of others [29]. As such, and in the frame of a mindful meditation, *Tong-len* combines an inner gesture of acceptance in which negative aspects are fully acknowledged while inhaling and beneficial aspects (e.g. ease of mind, kindness, etc.) are visualized to be given back to others while exhaling.

The *Metta* meditation can be considered as a precursor of *Tong-len* [30], as it predisposes the meditator to stay in contact with a source of inner warmth and to keep a kind attitude towards others. In general, meditations stemming from Buddhism, can be presented in a completely secular fashion, since they are directly concerned with the development of basic human attitudes, inner qualities and the resulting experiences that can be directly perceived and felt. Starting from the premise, that our mind is constantly designing our experiences and actions, it is believed that qualities like *Loving-Kindness* can be cultivated by everyone who has the wish to do so, transforming adverse attitudes such as indifference or aversion in the process.

First evidence has revealed that practicing *Metta* is linked to increased resilience and enhanced emotional regulation skills [31–33]. Furthermore, a recent study conducted with social workers during their professional training suggests that *Tong-len* might have strengthened their ability to transform even extreme distress encountered in contact with clients into a warm and caring prosocial motivation [34].

In sum, experts suggest that these methods can be strongly helpful in cultivating the motivation and mental strength to engage in compassionate behaviors towards others, especially by helping to overcome the barriers associated with one’s ability to be physically and emotionally present. Despite this promising evidence and although there are some existing contemplative approaches to end of life care, [35–37] there is a lack of empirical evidence regarding the effects of mindfulness and compassion-oriented meditation in palliative care in general. Specifically, of tailored made interventions that can help interdisciplinary palliative care teams; to jointly integrate the learned skills into their work daily routines.

Therefore, we designed, implemented and evaluated a program incorporating exercises drawn from the practices of mindfulness-, *Metta-,* and *Tong-len*, which were adapted to be taught in a secular fashion directly at the workplace of a palliative care unit. This approach also gave us the opportunity to observe the effects of bringing mindfulness and compassion-based meditation directly into the health care system context.

This pilot study had the following objectives: (1) to assess the feasibility of the intervention, (2) to make a first assessment of the potential effects and effect sizes concerning indicators of psychological distress, job situation and emotional regulation competences, (3) to explore the subjectively perceived impact of the training.

## Methods

### Design

Observational, pre-post pilot study with a mixed-method approach. Measurement points were at baseline immediately before the start of the 10-weeks intervention (t0) and after the intervention (t1).

### Participants

Participants were staff members of a palliative care center in a faith-based community hospital in Bonn, Germany, and were recruited by internal advertisements. The center includes an inpatient consultation unit, a liaison service, a home care service, a palliative care academy as well as a volunteer service. Since team development has been seen as integral to effective palliative care [1], staff members in all work areas were invited to participate. Each participant provided written and informed consent before being enrolled and anonymity of the analysed data was guaranteed. They were asked to fill out questionnaires before and after the training and to keep a home journal. Additionally, saliva samples were taken at pre-post to measure cortisol awakening response (CAR). Qualitative interviews with all participants were conducted after the intervention.

### Intervention

The intervention was designed and provided by an experienced meditation teacher and *Tong-len* expert (YUR). The intervention is a 10-week group program with four main objectives: 1) development of a mindful presence, 2) cultivation of *Loving-Kindness* 3) the practice of *Tong-len* in difficult situations and 4) the integration of those practices into daily work activities.

#### Practice elements

The intervention comprised a large variety of different practice elements (see Table 1) and consisted of an initial 2 h session and nine weekly practice days. On practice days, the staff members were offered the opportunity to participate in brief meditations sessions and one-to-one-sessions for a period of 4 h per shift. Participants were instructed to apply the learned techniques during work and to meditate daily at home. Additionally, they received a CD with guided meditation exercises for home practice. Posters reminding them to breathe or walk mindfully as well as posters containing *Metta*-sentences were distributed in the staff rooms. Participants were also encouraged to cultivate feelings of loving kindness using *Metta*-sentences when thoughts and feelings about patients and their relatives aroused during their free time.

**Table 1:** Key Practice elements of the training

Practice days at the hospital (8 h, 2 shifts); voluntary assistance to any session according to workload and schedule)	• Brief Meditation sessions in small groups every hour (15 Minutes). Announced by sounding a singing bowl in the ward. • One-to-one-sessions with the teacher on the half hour to discuss transfer (Max. 30 Minutes) • 3 sessions with all participants (1 h) at week 4, 6 and 10 to discuss embedding of practices in daily work-life and feedback.
Informal practice	• Mindful breaks • Walking Meditation • Mindful stops/ Practice anchors during daily work routines • *Metta*/*Tong-len* practice during patient /relative contact
Homework	• Meditation at home with CD • *Metta* Sentences during leisure time
Supporting material	• CD • Posters at the staff rooms

#### Course schedule

Table 2 gives an overview of the content of each session. In the initial 2 h session (week 1), mindfulness and compassion-oriented practices were introduced. In this session also the expectations and needs of the participants were explored via a Goal-Attainment-Scale (GAS). The application of the GAS scale had a twofold purpose: Firstly, the GAS scale served to evaluate the intervention fit to the needs of palliative care teams via the objective assessment of individual goals with respect to the intervention. Secondly, the application of the GAS scales helped to involve the participants actively in the study [38], by eliciting a common ground between participants’ expectations and the contents of the course. Goal suitability as well as observable and achievable improvement criteria was discussed with each participant by a trained psychologist and systemic counsellor (StS).

**Table 2:** Topic and content of each training session

Week	Topic	Summary of contents^a^
1	*Introduction*	Exploring the expectations and needs of the participants; setting of two individual goals for the intervention (GAS scale); introducing mindfulness and compassion-oriented meditations.
2	*Developing a mindful presence at work*	Learning how to stop: observing the breath, mindful breaks; finding individual practice anchors in daily routine (e.g. mindful stop before entering a patient’s room or before answering the phone, etc.); sitting meditation; walking meditation.
3	*Finding the inner source(s) of compassion*	Body awareness; preparation for the *Metta* Meditation: visualizing the inner source of compassion.
4	*Compassion-oriented Meditation in action*	*Metta* Meditation; how to apply compassion-oriented meditations at work/ during patient contact; *Metta-*sentences.
5, 6, 7, 8, 9 and 10	*Mindful presence in difficult situations*	How to apply *Tong-len* Meditation when confronted with suffering and difficult emotions.

^a^note that some contents, were repeatedly taught in more than 1 week

In the following 2 weeks of the program (week 2 and 3), a foundation was built for the introduction of compassion-oriented practices, by fostering the development of mindful presence and body awareness. The topic of week 4 was the Loving-Kindness Meditation. Week 5 to 10 focused on *Tong-len* practice.

Finally, although the meditation exercises were adapted to be taught in a secular fashion, participants could integrate their own spiritual resources if they wished to do so. A similar approach has been successfully implemented by Oman et al. [39]. In the study at hand, participants could apply the meditation instructions within the context of their own spiritual background (e.g. including a religious figure in the visualization of their sources of compassion) or without any such disposition at all (e.g. using neutral symbols such as light or color).

### Data collection

Study packages were distributed in staff mailboxes of the respective participants before and after the training. They included the study questionnaire and 4 cotton swabs for saliva sampling. Prior to the training, participants received an information package containing an introductory letter as well as an instruction sheet for the performance of the saliva sampling. Participants filled the questionnaires at home and returned them in a closed envelope together with the salivary samples at the introductory session and at the time of the qualitative interviews, respectively.

Semi-structured interviews were conducted with all participants by the first author to gain insight into the subjective experience of the training. The interview questions were developed by the first author under the supervision of a qualitative research expert (See Table 3 for guiding questions). Staff members were interviewed individually by the first author during working hours in a private room at the palliative care unit. At the end of the interview, we conducted an additional short interview to obtain the level of goal attainment. Interview durations ranged from 27 min to one and a half hour (SD = 52 min).

**Table 3:** Guiding questions

Primary Focus	Inquiries
Part I – Experiences with the program Introductory narration stimulus: I would like to ask you to describe your experiences with the program just as they occurred for you personally, from the moment, you heard about it. I am interested in any details that appear important to you. Take your time. I will not interrupt you at the beginning.	
Probes	You mentioned before that… could you describe that in more detail? / Could you tell me more about that? You mentioned before that… What do you think caused this development? / Could you tell me a story that illustrates this aspect?
Part II – Motivation, Outcomes and Integration into work life 1. Motivation 2. Training Outcomes 3-6 Course Implementation	1. What motivated you to participate in the program? 2. Did you notice any changes in your self-care behavior / contact with patients or colleagues / when dealing with difficult situations, through the course of the program? 3. What have been your experiences with *Metta / Tong-len*? 4. Which one of the program elements was most accessible to you? 5. Which one of the program elements did you find most difficult to relate to? 6. Was there anything in the training that irritated you /seemed unfamiliar to you /surprised you?

### Measures

#### Self-report instruments

*Maslach Burnout Inventory (MBI)* [40]. The MBI for the Human services consists of 22 items scoring on three subscales: emotional exhaustion (9 items), depersonalization (5 items) and personal accomplishment (8 items). With regard to the psychometric properties of the German version, reliabilities are α = 0,82 for emotional exhaustion, α = 0,67 for depersonalization and α = 0,75 for personal accomplishment. The validity could be also confirmed for the German version [41].

*Perceived Stress Questionnaire (PSQ-20)* [42]. The PSQ-20 is a 20 items inventory that assesses perceived stress on the subscales worries, tension, joy (reversed scale) and one stressor dimension. A total score can be computed from all items. The PSQ has been demonstrated to be highly correlated to Quality of life measures and physiological stress markers. Cronbach’s alpha for the total score of the German version is α = 0.85 (*n* = 650). Its sensitivity to change has been also verified. [43].

*Hospital Anxiety and Depression Scale (HADS-D)* [44]. Consisting of 2 Subscales of 7 items each*,* the HADS-D is a frequently employed instrument for screening anxiety and depression. The psychometric properties of the German version (*n* = 6200) can be considered as satisfactory. The reliability for the anxiety subscale is α =0.80 and for the depression subscale α = 0.81, respectively. Convergent Validity has been demonstrated for the HADS-D through well documented correlations with related measures [45].

*The Symptom Checklist-90-R, Somatization Scale (SCL-90-R-SOMS)* [46]. In order to assess somatic complains the somatization 12-item subscale of this well-established instrument for the evaluation of psychopathology symptoms, was applied. The reliability of the SOMS scale for the German version ranges from α = 0.70 - 0.87. The SOMS scale has also displayed positive associations with instruments measuring global health status [47].

*Emotion Regulation Skills Questionnaire (ERSQ-27)* [48]. The ERSQ is a 27-item instrument that evaluates 9 emotion-regulation skills with three items each and a total score. A positive correlation between these skills and mental health, as well as a negative association with measures of psychopathology and emotion regulation deficit has been shown in multiple samples [49, 50]. The reliability values for the ERSQ are adequate to good (α = .90 for the total score and α = .68 -.81 for the subscales).

*Work Situation (NRS).* Changes in the perceived job situation were assessed on the dimensions work satisfaction, enforcement through work and work enjoyment by the use of 3 numeric rating scales asking how satisfied participants were with their current work situation, how strengthened they felt by their work and how much joy they experienced at work. The Items were based on a rating scale ranging from 0 (not at all) to 10 (very much).

*Goal attainment scaling* (GAS). GAS have been widely employed in a variety of settings, including the assessment of mindfulness interventions, to assess program impact [51]. GAS include a goal chart, containing up to five different levels of attainment reaching from −2 (far less than expected) to +2 (far more than expected) with 0 referring to adequate goal attainment. The level of goal attainment was assessed at the end of the training, through a short interview conducted by the first author.

#### Cortisol

Before and after the program, participants were asked to collect 4 saliva samples (0, 10, 20 and 30 min. After waking up from sleep). Salivary cortisol concentrations were measured by the Laboratory of Dirk H. Hellhammer, Trier University, Germany. The samples were centrifuged at 2000 g for 10 min and cortisol levels (nmol/l) were analysed using a time–resolved fluorescence immunoassay with fluorometric end point detection (DELFIA) [52].

Based on the four assessments, the area under the concentration time curve (AUC) was calculated as an indicator of cortisol awakening response. Three variables were obtained: *AUC total*, i.e. the total amount of cortisol under the curve; *AUC basal*, denoting the initial amount of cortisol secretion over time; and *AUC net,* describing the difference between AUC total and AUC basal [53].

#### Feasibility data

Feasibility was assessed employing program attendance lists. Additionally, participants recorded the frequency and duration of practice at home. Satisfaction with the course was evaluated utilizing a self-constructed questionnaire.

#### Qualitative analysis

Semi-structured interviews were conducted with all participants to gain insight into subjectively perceived outcome areas, and in order to reveal potential links between outcomes and training elements (See Table 2 for guiding questions). Further areas explored were integration into daily work life, motivation to participate, work related resources and distressing aspects, as well as the interplay between compassion and personal wellbeing. In relation to the objectives of this paper, only the results concerning the effectiveness of the program are presented here. Interview durations ranged from 27 min to one and a half hour (SD = 52 min). The audiotaped interviews were transcribed including pauses and intonation and analysed employing the technique of the integrative interview analysis (Ger. Integrative Basisverfahren), which integrates grounded theory (GT), ethno methodological conversation analysis methods, as well as Manheim’s documentary method, in a reconstructive-hermeneutic analysis process [54]. In this process, meaning is reconstructed based on the analysis of linguistic phenomena, communication patterns when addressing topics (Ger. Thematisierungsregeln) as well as interactional and argumentative aspects (e.g. how the interviewees position themselves).

These information sources are condensed into motives (Motive) before being grouped into central motives (zentrale Motive) which are central meaning figures. First, this is carried out in the frame of a sequential and detailed reconstructive analysis of individual cases before a comparison process between the interviews take place. Central motives are then similar to the central categories in GT, as they describe a core category around which all other categories group [55]. The difference however resides in the application of more than one analytical approach during the coding process, as well as in the comparison step, taking place after analysing individual cases and not from the beginning of the coding process, as it is performed in GT [56]. Therefore, we employ the original terms of central motives and motives, instead of the more common terms of categories and themes in order to describe our results. The term motive is not to be understood in a psychological sense as the motive behind an action but as a pictorial motif in a semantical sense. A motive is formed by the impressions given by the informants on multiple levels of textual analysis. In this sense, a central motive is a meaning Gestalt expressed consistently through the data, and formed by a coherent set of linguistic and communicative patterns [55]. This implies that motives are often presented with a rather descriptive wording since they should transport a topic like a picture. Motives can be for example metaphors, figures of speech or discursive elements that refer to a central topic. See for example [57].

Interviews were analysed by an interpretation group consisting of seven members. Six of them were psychologists working at the research department where this study was planned and conducted (see first author affiliation). The group was led by a sociologist (AA) who has not otherwise collaborated with the research team of the study.

Besides the differences mentioned above, the coding procedure of this method is analogue to the GT coding procedure of open coding, axial coding and selective coding [57]. First, group members identified sensitizing concepts in passage-by-passage discussions of 11 transcripts. We then prepared a detailed case summary of each of these 11 interviews, which led to main motives. The emerging motives were used by the first author (CO-R) and a second member of the analysis group to structure the interviews while allowing new motives to be added. These were iteratively discussed in the group and further developed if necessary before a final interpretation of motives and a condensation into central motives took place.

### Statistical analysis

T-tests for dependent samples were employed to test for differences between baseline and post-intervention. Effect size Cohen’s d was computed from t-values by the formula d = t_c_[2(1 - r)/n]^1/2^ as recommended for paired samples [58]. Significance level was set at *p* = 0.05. Descriptive analyses were conducted on the socio-demographic variables of age, gender and profession and on all quantitative variables to obtain means, standard deviations and percentages. All analyses were conducted using SPSS 22.

## Results

A total of 85% (28 from 33) of the total population of the palliative care staff engaged in the program. Twenty-eight staff members were enrolled, with one discontinuing training after the first week. All other 27 participants participated in the course regularly and 26 of them returned post-intervention assessments. Socio-demographic characteristics of the sample are provided in Table 4.

**Table 4:** Socio-demographics (*n* = 28)

Variable	Results
Mean Age (years), ± SD, Range	46.4 ± 5.8 (Range 37-57)
Gender	
Male	*N* = 7 (25%)
Female	*N* = 21(75%)
Profession (frequencies)	
Nurses	19
Physicians	1
Social workers	2
Psychologists	1
Physiotherapists	1
Administration	3
Volunteer	1

### Self-report scales

All self-report measurements are displayed in Table 5. Baseline measures for burnout, anxiety and depression were below cut-offs, indicating a mild burden among the participants. Nevertheless, significant reductions in anxiety and in the burnout component emotional exhaustion could be found. Additionally the burnout component personal accomplishment increased significantly after the training. No significant differences were found for somatization and depression. Regarding stress, the total PSQ score as well as the scores of the PSQ subscales worries, tension and demands were significantly reduced after the intervention. Furthermore, participants demonstrated a heightened level of general joy, as measured by the corresponding PSQ subscale.

**Table 5:** Means, standard deviations (SD), t-values and degrees of freedom (df) of the self-report variables MBI, PSQ-20, HADS-D, SCL-90-R-SOMS, SEK-27 and work situation, as well as cortisol measures. (*p*-values are for paired sample t-tests, effect sizes refer to pre-post changes)

	Before the training		After the training		t (df)	p*	Cohen’s d
	Mean	SD	Mean	SD			
MBI (Burnout)							
Exhaustion	14.85	9.07	11.29	7.63	−3.13 (24)	.005**	0.41
Depersonalization	2.72	2.85	2.53	2.80	0.71 (24)	.48	0.07
Personal							
Accomplishment	39.27	4.88	41.22	4.03	−2.71 (24)	.012*	0.43
PSQ (Stress)							
Worries	.23	.14	.17	.11	2.39 (25)	.025*	0.48
Tension	.39	.22	.26	.14	3.51 (25)	.002**	0.69
Joy	.65	.21	.73	.19	−2.25 (25)	.033*	0.38
Demands	.43	.23	.36	.16	2.20 (25)	.037*	0.33
Total	.35	.18	.27	.12	3.08 (25)	.005**	0.52
HADS-D (Anxiety and Depression)							
Anxiety	5.42	2.66	4.31	3.04	2.45 (25)	.022*	0.39
Depression	3.31	2.21	2.77	2.14	1.59 (25)	.12	0.25
SCL (t-score)							
Somatization	49.31	8.94	47.50	8.58	1.17 (25)	.26	0.21
Emotion Regulation Skills Questionnaire (ERSQ-27)							
Awareness	3.00	0.77	3.32	0.49	−2.87 (24)	.008**	0.45
Clarity	3.17	0.74	3.27	0.61	−1.01 (24)	.32	0.15
Sensations	3.08	0.78	3.12	0.59	−0.24 (24)	.82	0.06
Understanding	3.25	0.73	3.23	0.63	0.26 (24)	.80	0.04
Acceptance	3.07	0.83	3.16	0.65	−0.76 (24)	.45	0.12
Resilience	2.77	0.97	3.16	0.80	−2.47 (24)	.021*	0.43
Self-support	2.87	0.92	3.01	0.77	−0.96 (24)	.35	0.17
Readiness to							
Confront	2.83	0.71	2.90	0.69	−0.76 (24)	.46	0.11
Regulation	2.73	0.89	2.90	0.65	−1.57 (24)	.13	0.20
Total	2.97	0.66	3.12	0.55	−1.85 (24)	.08	0.24
Work situation							
Satisfaction	7.75	1.34	8.19	1.47	−1.63 (25)	.117	0.31
Enforcement	7.83	1.81	8.15	1.46	−1.12 (25)	.274	0.2
Enjoyment	7.62	1.44	8.35	1.06	−3.06 (25)	.005**	0.6
CAR, nmol/l							
AUC total	333.68	106.21	367.68	214.97	0.86 (23)	.396	0.19
AUC basal	250.21	103.07	292.15	117.40	−1.01 (23)	.323	0.26
AUC netto	83.47	93.58	75.52	111.69	0.25 (23)	.806	0.08

* *p* = 0.05; ** *p* = 0.01

Regarding work situation, the variable work enjoyment measured via a numeric rating scale, also increased significantly with training. In the area of emotion regulation, two variables, namely *awareness* and *resilience* (ability to deal with difficult emotions*)* showed significant and substantial improvement.

### Cortisol (Table 5)

Cortisol variables did not change significantly during the course of the program.

#### Goal attainment scaling

**Fig. 1:**
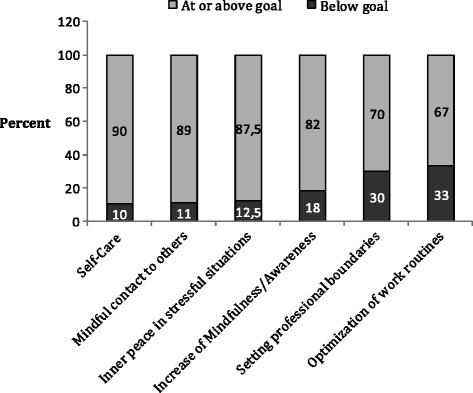
Goal categories and the level of their attainment success in percent

Overall, 85% of the individual goals were attained after the intervention with *self-care* goals having the highest and *optimization of work routines,* the lowest level of attainment (see Fig. 1). Attainment success was of 18% for goals being reached far better than expected (+2); 41% better than expected (+1); 26% to the expected level (0); 14% of the goals were not reached at the expected level (−1) and 2% did far worse than expected. Participants’ goals were grouped into six categories using summative techniques for coding qualitative data. With exception of the category optimization of work routines, which made a 6% of the total goals, the frequency of occurrence was similar for every goal group and varied from 16% (*inner peace in stressful situations*) up to 22% (*increase of mindfulness*).

### Qualitative data

Altogether, seven central motives with a total of 18 motives regarding the perceived main effects of the training could be identified (see Table 6). A benefit of the training mentioned in every interview was an *empowerment of self-care* behaviors and attitudes. Additionally, participants felt that the training helped them to effectively incorporate *mindful pauses* in their daily work routines, especially during tense and difficult situations. A reduction in *rumination,* especially regarding *work-related thoughts during leisure time*, was also noted by the participants. Some staff members also identified a reduction of *empathic distress indicators* and strengthening of *interpersonal connection skills.* An enrichment of *team communication* and *conflict management* could also be identified by two of the team leaders. One central communication pattern was the determined *self-positioning of the participants as having already a compassionate attitude* in their work before the intervention. The staff members repeatedly wanted to underpin their professional competence in this area. Although this information was not explicitly requested, participants very often referred to their professional expertise in dealing with suffering, for example by naming the years of labor in the field, or their educational background. They also drew very clear lines to other areas in health care regarding attitudes in health care, or offered examples of how compassion already formed part of their work. However, some staff members described that the program supported different aspects of the relationship building process.

**Table 6:** Reported outcomes: motives and central motives regarding the perceived effects of the training

Self-care Empowerment 1. Enhancing feelings of self-efficacy 2. Allowing self-care during leisure time. 3. Stepping out the hamster wheel	1. “Well, I just consciously brought the sole of my feet in contact with the ground, one by one, and this was instantaneously soothing, as if one is caressing oneself...It was such a sense of self-efficacy: I was able to easily do something good for myself.” (Person 14) 2. “I did some things more consciously, whether being out in the garden or doing sports. Just for myself. These things suddenly had a different quality to me.” (Person 01) 3. “I recognized this as a quality of its own, becoming aware of the fact that I need time and space in order to get in contact with myself and if I don’t find the time to just do a short mindful stop or to walk mindfully once in a while, then chances of establishing a good connection with the patients are low…and it doesn’t even take that much. Evidently, short time spans suffice.” (Person 21)
Mindful pauses in the midst of stress (Ger. Innehalten)*	1. “I stopped just for a short moment in very chaotic or critical situations and got myself out of that sort of vortex we can very quickly get in to here, where you start thinking all this is so horrible and awful and too much and when you take a step back, you notice it’s not necessary to take it upon you. And then the situation sort of widens and changes. I did that all the time.” (Person 05) *The name of this central motive poses a difficult translation situation. There is no English term that truly corresponds to the German expression of *Innehalten.* Innehalten implies shortly stopping in time in a conscious way. Participants referred to this skill as Innehalten in difficult or stressful situations which represent a specific training effect. Therefore we choose this descriptive translation as it transports better the quality of this effect.
Reduction of Rumination 1. Interrupting rumination spirals at work 2-3. Reduction of work-related thoughts after work (cognitive irritability).	1. “I′m a person who really worries an awful lot…and I am now more capable to interrupt this at an earlier stage: to say make a cut and see what good you can do in this very moment, and how you can change the situation towards something positive.” (Person 18) 2. “On my holiday I noticed very clearly that sending out compassion and practicing Tong-len for others, helped me to stop rumination on work. For 14 days I thought very little about it. Before, I often had difficulties to just call it a day and relax.” (Person 19) 3. “I tried the suggestion made in the training to formulate these, I would call them intercessions** for patients at home and this was great for me. I had the feeling, that in the moment I consciously went again into the problems of the people, wishing them well, I was liberated. That was rather surprising for me because when I first heard about it, my first thought was: in my free time I don’t want to deal with patients issues anymore, rather I want to distract myself. But then I noticed that distraction doesn’t really work. It is like a covering up. By doing this, I didn’t cover things up but handed them over.” (Person 10) ** means ***Metta*** **-sentences**
Reduction of empathic distress 1. Positive Re-appraisal of difficult care situations 2. Transforming difficult emotions 3. Reducing negative involvement with suffering	1. “I find dying situations less scary. I mean, nobody knows what is happening during death. It is something you cannot grasp and that can be frightening… I have had patients experiencing anxiety and unpleasant things. This [Metta] helps you to overcome those situations. It helps you to see the good and positive sides of it, to say O.K., accompanying them in such a situation can also be very beautiful.” (Person 18) 2. “We had, how should I put it, a very satisfied patient and a very overstrained wife, who was latently accusing every time she got in contact with the staff. Nothing we did was good enough for her…There is always a danger of taking these things personally: «you are not kind to my husband» or «you’re not doing a proper job ». In the past, I would have reacted defiantly, not towards the patient, but with the relatives. Now, before I drove to work, I consciously decided to encounter that with something positive, something warm. That allowed me to still create a warm atmosphere. This was very important for me.” (Person 09). 3. “I felt less responsible. I could be present with more equanimity. Well it wasn’t like calming down already but I didn’t feel like being called upon to change the situation by any means.” (Person 25)
Enhancing interpersonal connection skills 1. Being present with suffering 2. Connecting to the patient as a person. 3. Taking a bold step onto closeness 4-5. Embodying compassionate feelings	1. “I can recount some emotionally loaded situations, either because it was a difficult topic or because the other person was sad, or they were difficult for me, because of how stressed I felt, by having this feeling, I just can’t engage in the situation because there are so many other things that I have to do. Then, I brought my attention to the breath and tried the heart visualization and in two occasions I could strongly experience how relieving this was. I mean, I could develop compassion then, and stay in the situation. This was very beautiful for me.” (Person 23) 2. “I had one situation with a patient who is strongly anxiety-affected because she is leaving her youngest son and she is not sure yet how things will unfold, who is going to take care of him and so on. And although she took care of everything there is nothing there she can rely on with absolute certainty. I found that was a challenging setting of conversation. Then I tried to practice Tong-len… And I left the conversation more satisfied. I had the feeling I gave everything I got. I mean not only on a counseling level, but also on an interpersonal level.” (Person 25) 3. “There was a patient who was already in the dying phase…His family was also there. Somehow they were already waiting for him to die and remained quite distant and tense. I had the feeling something was wrong, that he needed something else. He didn’t need people sitting around and watching him in awe. In that moment I put my hand in his heart area and then it came out of me. This warmth, a feeling which I can’t really describe and which I could give to the patient. It was like saying to him: - everybody is here, everything is fine … you can let go. I expressed my gratitude to him and there was so much pouring out of me which I sent out to him. I said it is fine, you can let go and then he died…The relatives came to me afterwards asking –“what was that? Everything happened so quietly and it was a wonderful atmosphere.” (Person 18) 4. “That is the practice [Metta] I liked the most… because it was very concrete and I could visualize it here [touches the heart area]. This really did me very good. I find it interesting how this area immediately gets very warm…it was thrilling for me, to see my source of strength. I mean this is something I already know. I know the sources of my strength, but with the meditation I could localize them and give them a name.” (Person 22) 5. “During one interaction in particular I could realize how good this works. Simply by knowing, that through being present in my body, through becoming aware of my body I can irradiate so much and on the same time be able of receiving suffering. That just felt so good.” (Person 14)
Team Communication 1. Speaking openly about distress 2. Finding common ground on self-care issues 3. Dealing with conflicts in team	1. “I didn’t anticipate such openness, that everyone would participate so open mindedly. I wouldn’t have expected that so many people here have the same kind of problems... To realize, that ultimately all feel very similar to oneself. I found that very impressing, surprising and beautiful, to realize that one is not alone.” (Person 19) 2. “During one supervision session we agreed on the fact that our lunch break is not really a break. We declared that during this half an hour we don’t want to talk about patients. This certainly was an aspect fostered by the training. Otherwise it wouldn’t have been brought to light in this way.” (Person 19) 3. “I recently witnessed a tense situation between two team members. I thought, well this is going to be the usual old fighting and I perceived, that at some point they looked at each other smiling, saying ok let’s breathe deeply for a moment and there was a change of outlook and somehow the whole energy changed.” (Person 15)
Self-positioning as compassionate 1-2. Referring to professional expertise 3. Offering examples of compassionate work 4. Setting Ingroup /Outgroup boundaries to other areas in health care	1. “Being compassionate to the patients, conveying compassion and being there for them, is very clearly our task here. However, seeing this task from the perspective of those meditations made it easier for me.” (Person 14) 2. “I just finished my education as a grief counselor 1 year ago…As I just said, communication skills are integral part of our work.” (Person 27) 3. “I believe this is a capability to love you carry within you, in terms of loving not others, but persons. That doesn’t mean you must like everything they do. Love is the strongest among all positive feelings that we can have in ourselves. To come to the point where it can flow freely and you can stand up for it, is a process. Eventually this became natural for me. Not only to feel it but to show it. I mean, that’s what people confirm to me, and this touches me so deeply when patients say, Thank you very much! You did that very well! and I think to myself, I just washed them and bedded them, but there was probably something else beyond that, that caused them to notice: somebody is there for me, radiating calmness and presence…and that’s somehow compassion too.” (Person 01) 4. “I internalized this attitude only until I started working at the palliative care center. At the intensive care unit, when relatives were irritated, one just used to say they are annoying. Here you handle this in a total different way. You think to yourself they are worried or they feel insecure.” (Person 24)

### Feasibility data

#### Compliance

The average time per week, during which participants saw the mindfulness teacher was 32 min (SD = 8.23). On practice days, most of the participants attended one or two meditation sessions per day. Due to alternating work-shifts and a varying amount of working time, not every participant worked on practice days; although they often came to attend the training during leisure time. A total of 57% of the participants attended at least 6 of the 9 practice days plus the introductory session, which in turn results in a compliance rate of 70%. Home journals (*n* = 20) yielded a mean frequency of practice of 3 times per week and a total time of exercise of 30 min per week.

#### Satisfaction with intervention

Three quarters of participants were satisfied with the course (60% = very satisfied, 16% = satisfied). While none expressed dissatisfaction, 24% of them, were only partly satisfied. In addition, 96% declared that they plan to implement these techniques into their work in the future and 88% would recommend the course to other palliative care professionals.

## Discussion

To our knowledge, this is the first study that assessed an “on the job” program including mindfulness and compassion-based meditation for palliative care teams, utilizing a mixed-method approach. Overall, quantitative and qualitative data provides first evidence on the feasibility and potential benefit of the training in reducing clinicians distress, and fostering a variety of resilience aspects.

Concerning *distress,* quantitative data suggest a reduction in perceived stress, anxiety and burnout, with effect sizes ranging from Cohen’s d = 0.39 – 0.69. Values in all variables were low at pretreatment making these effects even more supportive for the intervention. These findings are in line with two reviews summarizing the benefits of mindfulness among healthcare practitioners [23, 59] and resemble the medium range effect size (d = 0.50) identified by Grossman et al. in their meta-analysis regarding the efficacy of mindfulness based interventions on mental health variables [60].

A possible mechanism of the training is an increase of awareness. According to a recent survey of palliative care practitioners (*n* = 387) higher levels of self-awareness go hand in hand with lower levels of burnout and compassion fatigue [61]. Our qualitative data suggest that awareness was fostered in a variety of situations. For example, by bringing mindfulness practice into personal activities, by mindful pausing during work routines or by intensifying mindfulness during the patient contact. GAS Scale assessment also revealed that personal goals regarding an increase of mindfulness and awareness were reached in 82% of the time. This could be an explanation for the reduction of psychological distress found in this study.

Recent evidence also supports the role of awareness as an important pathway for fostering self-care in palliative care practitioners [61]. Consequently, several participants felt that the training directly improved their self-care efficacy and empowered them to implement self-care behaviors. Furthermore, balancing self and others interests is important for self-care [62]. From the beginning, the participants were instructed to focus their compassionate feelings at first towards themselves before extending to others. Quantitatively, this is mirrored in the fact that the individual goal categories regarding self-care behavior and mindful contact to others were reached to a high percent and almost equally, suggesting training specificity.

Existing evidence also suggest that practicing *Metta* meditation can strongly increase personal resources with self-generated positive emotions being the central mechanism of change [31]. Accordingly, the work enjoyment measured in this evaluation was significantly higher after the training.

Physiologically, there were no significant changes in cortisol levels. However, results regarding the impact of mindfulness-based interventions on CAR are mixed [63–66]. Additionally, since 70% of our sample consisted of nurses with varying work shifts, CAR may have been affected by confounding variables like the diurnal rhythm [67, 68]. Considering the incongruence of this result with the effectiveness of the intervention yielded in the self-report scales, GAS and interview data, our results questions the suitability of CAR as an indicator of distress for healthcare providers.

Qualitative exploration of *distress* also indicated an improvement in relevant areas. A novel finding is the perceived link between practicing *Metta* meditation and a perceived reduction of cognitive irritability. Cognitive irritability describes a state of mental stress resulting from perceived discrepancy between a given situation at work and an important personal goal. Hereby work strain is manifested during leisure time in form of ruminative efforts concerning goal-achievement that can hinder psychological regeneration and be a risk factor for depression [69]. Correspondingly, a newly published study exploring training needs among palliative care practitioners revealed that there is a necessity for strategies to help reduce ruminative thoughts and negative self-talk [27]. This result highlights the potential of *Metta* meditation in targeting distress sources particular to palliative care.

Two of the team leaders, also felt the training enhanced team communication concerning self-disclosure, mutual consent on self-care issues and conflict management.

Finally, qualitative results suggest that as already proposed by Singer and colleagues [19], compassion-oriented meditation can reduce distress generated in the patient contact and foster interpersonal connectedness.

Since emotional regulation plays a crucial role in the empathic response, [70] we assume the significant improvement of two emotional regulation skills: i.e. awareness of own emotions and the ability to deal with difficult emotions, to be a further central mechanism of action.

It should be noted, that none of the participants stated the training would have enhanced their compassion in general. Furthermore, a core rule of addressing topics in the qualitative interviews was a self-positioning of participants as having already a compassionate attitude in their work. Despite not being asked directly, they inferred in different ways to their competence as compassionate caregivers. For example, naming the exact number of years of professional experience, setting Ingroup/Outgroup boundaries to other areas in health care concerning attitudes to care, or offering examples of how they act compassionately in their work. This is not surprising, since learning to rest in the experience of suffering and developing healing connections are key aspects of palliative care [71, 72]. This finding leads to the question of how compassion can be developed in the case of health care providers and is also in consonance with the research work on healthcare compassion published recently by Sinclair and colleagues [6, 73, 74]. They concluded that patient’s perceived compassion as being rooted in the virtues of their health care provider, generating the first evidence-based definition of compassion—*a virtuous response that seeks to address the suffering and needs of a person through relational understanding and action*. The authors note however that, compassion qualities can be cultivated or eroded over time, with practice setting being one of the most powerful modifiers. Thus, although the roots of compassion seem to be innate, current evidence suggests that training, including meditation, can help to sustain compassion and nurture it over time. Accordingly, participants in this study recognized the usefulness of the course and reported multifaceted benefits.

Concerning the implementation of the intervention, the program proved to be feasible and satisfactory for staff members in a busy palliative care unit. A natural affinity between mindfulness practices and palliative care has been described before [75]. This compatibility could explain why the team members, mostly novice meditators, received the training with great receptiveness.

Though the results of this study are encouraging, several limitations should be take into account when interpreting the data. First, it should be borne in mind, that a training duration of 5 weeks for *Metta* and *Tong-len* might have been too short for the practices to unfold their full potential. Thus, future evaluations should consider a longer training duration and adding guided, larger practice spans, outside working hours in order to assure in depth practice. In fact, this was a wish expressed by all participants at the feedback round.

Second, due to the lack of a control group effects cannot be causally assigned to the intervention. In addition the small sample size limits statistical power and generalizability. Furthermore, the reported effects may be influenced by the attention provided by the evaluators or by an organizational focus on self-care issues at work. Thus, further studies with higher internal validity are needed in a next step. However, the aim of this study was to assess feasibility and implementation of this rather unconventional intervention. The advantage of our approach is a high ecological validity, since participants had the opportunity to integrate the practices in real-life situations and person-to-person interactions (cf. [76]). Ecological validity describes the extent to which research findings are achieved through methods (e.g. treatment) that are representative of events that occur in daily life and thus, can be generalized to real life settings [77].

In conclusion, our results provide important initial support regarding the usefulness of this newly developed *on-the-job* intervention for palliative care teams.

## Conclusions

Although the work in palliative care can be highly rewarding, there are inherent stressors that can affect the wellbeing of palliative care practitioners. Furthermore, there is a necessity for less protection oriented self-care strategies that allow practitioners to still encounter the suffering they are confronted with, with an open and kind attitude. Even though there is emerging evidence for the potential of compassion-oriented practices, there is a lack of applied research investigating the effects of these practices under real work conditions. We aimed to address this gap and elucidate how mindfulness and compassion-oriented practices can be implemented within the clinical setting. Although we could not find an enhancement of compassion in general, participants reported a benefit from the training in the areas of self-care, emotional regulation skills, work-related distress, mindfulness at work and interpersonal connection skills, suggesting that highly compassionate individuals benefit differently from these practices. Qualitative interviews also suggest, that based on their expertise, staff members addressed areas they saw as relevant in order to foster sustainability of their compassionate behaviors at work. This interplay between compassion and wellbeing should be investigated more deeply in order to determine the role of self-care in nurturing compassionate behaviors in health care. Thus our study contributes to a more targeted implementation of mindfulness and compassion-oriented practices in clinical settings, that can be beneficial for both practitioners and their patients.

## Abbreviations

AUC: area under the curve
CAR: Cortisol Awakening Response
DELFIA: dissociation-enhanced lanthanide fluorescence immunoassay
ERSQ-27: Emotion Regulation Skills Questionnaire
GAS: Goal attainment scaling
GT: Grounded Theory
HADS-D: Hospital Anxiety and Depression Scale
MBI: Maslach Burnout Inventory
NRS: Numeric Rating Scales
PSQ-20: Perceived Stress Questionnaire
SPSS 22: Statistical Package for the Social Sciences
